# Electron Paramagnetic Resonance (EPR) Spectroscopy in Studies of the Protective Effects of 24-Epibrasinoide and Selenium against Zearalenone-Stimulation of the Oxidative Stress in Germinating Grains of Wheat

**DOI:** 10.3390/toxins9060178

**Published:** 2017-05-27

**Authors:** Maria Filek, Maria Łabanowska, Magdalena Kurdziel, Apolonia Sieprawska

**Affiliations:** 1 Polish Academy of Sciences, The Franciszek Górski Institute of Plant Physiology, Niezapominajek 21, 30-239 Cracow, Poland; 2 Faculty of Chemistry, Jagiellonian University, Ingardena 3, 30-060 Cracow, Poland; labanows@chemia.uj.edu.pl (M.Ł.); kurdziel@chemia.uj.edu.pl (M.K.); 3 Institute of Biology, Pedagogical University, Podchorążych 2, 30-084 Cracow, Poland; apolonia.sieprawska@gmail.com

**Keywords:** zearalenone, oxidative stress, detoxification, brassinosteroids, selenium, wheat, EPR

## Abstract

These studies concentrate on the possibility of using selenium ions and/or 24-epibrassinolide at non-toxic levels as protectors of wheat plants against zearalenone, which is a common and widespread mycotoxin. Analysis using the UHPLC-MS technique allowed for identification of grains having the stress-tolerant and stress-sensitive wheat genotype. When germinating in the presence of 30 µM of zearalenone, this mycotoxin can accumulate in both grains and hypocotyls germinating from these grains. Selenium ions (10 µM) and 24-epibrassinolide (0.1 µM) introduced together with zearalenone decreased the uptake of zearalenone from about 295 to 200 ng/g and from about 350 to 300 ng/g in the grains of tolerant and sensitive genotypes, respectively. As a consequence, this also resulted in a reduction in the uptake of zearalenone from about 100 to 80 ng/g and from about 155 to 128 ng/g in the hypocotyls from the germinated grains of tolerant and sensitive wheat, respectively. In the mechanism of protection against the zearalenone-induced oxidative stress, the antioxidative enzymes—mainly superoxide dismutase (SOD) and catalase (CAT)—were engaged, especially in the sensitive genotype. Electron paramagnetic resonance (EPR) studies allowed for a description of the chemical character of the long-lived organic radicals formed in biomolecular structures which are able to stabilize electrons released from reactive oxygen species as well as the changes in the status of transition paramagnetic metal ions. The presence of zearalenone drastically decreased the amount of paramagnetic metal ions—mainly Mn(II) and Fe(III)—bonded in the organic matrix. This effect was particularly found in the sensitive genotype, in which these species were found at a smaller level. The protective effect of selenium ions and 24-epibrassinolide originated from their ability to inhibit the destruction of biomolecules by reactive oxygen species. An increased ability to defend biomolecules against zearalenone action was observed for 24-epibrassinolide.

## 1. Introduction

One of the main active oestrogenic mycotoxins produced as a secondary metabolite by the pathogens of the *Fusarium* groups is zearalenone (6-(10-hydroxy-6-oxo-*trans*-1-undecenyl)-*β*-resorcyclic acid lactone; ZEA). Plants accumulate ZEA in varying degrees, depending on the genotype, atmospheric conditions, and region of cultivation. However, extensive data exists that indicates the contamination of cereals with this mycotoxin on a global scale; as a consequence, food products obtained from them could also be potentially contaminated [1,2,3,4]. It has been demonstrated that stressogenic action of this toxin in plants is several times stronger than that of naturally occurring oestrogens, and causes a significant decrease in the germination of seeds [5], leaf damage, and reduced yield [6,7]. Studies have also shown relationships between an increase in the ZEA level in animal tissues and the disease frequency [8].

Detoxification strategies to reduce or eliminate the toxicity of ZEA by chemical, physical, and biological methods were suggested as being crucial to improve food safety and reclaim contaminated products [4,9]. Total or almost-total degradation of ZEA was obtained during ozone [10] and H_2_O_2_ [11] treatment. However, using such a reactive oxygen species can itself induce the stressogenic reactions in cereal grains as an effect of oxidative stress action, and cause the modification of their biochemical composition [12]. The group of reactive oxygen species (ROS) generated in oxidative stress conditions is comprised mainly of ^1^O_2_ (singlet oxygen), H_2_O_2_ (hydrogen peroxide), O^•−^_2_ (superoxide radical), and OH^•^ (hydroxyl radical) species. A quantitative increase in free oxygen radicals is regarded as a factor directly responsible for damage of protein, DNA, and lipids. Thus, this affects normal cellular functioning [13,14]. Redox homeostasis in cells is restored by the antioxidants from groups of enzymes such as the superoxide dismutases (SOD), peroxidases (ascorbate-APX and guaiacol-GPX) and catalase (CAT). There are also antioxidants from non-enzymatic molecules, such as ascorbic acid (AA), reduced glutathione (GSH), α-tocopherol, carotenoids, phenolics, flavonoids, and proline [15,16,17]. On the basis of results obtained using electron paramagnetic resonance (EPR) spectroscopy, another approach to the natural “deactivation” of ROS in plant tissues was found [18,19]. EPR studies of the biological materials indicated that organic molecules such as carbohydrates and proteins may stabilize the excess electrons generated in stress conditions by the formation of stable long-lived organic radicals [20]. The EPR method allows for a description of such radical species, as well as of the stress-induced chemical changes occurring in the environment of paramagnetic ions. These ions occupy the active centers in proteins, which are mainly enzymes [21]. It was found that changes in the coordination sphere of paramagnetic metal ions taking place in stress conditions depended on plant genotypes, and it has been postulated as a sensitive indicator in the classification of the tolerance of different genotypes [19].

In this study, we proposed that using selenium (Se) ions and a steroid (brassinosteroid) as chemicals in low concentrations may be able to decrease the stressogenic action of ROS induced by ZEA treatment of wheat grains. Protective properties of Se against oxidative stress were confirmed in numerous studies, having been stimulated by different physical and chemical factors [22]. Selenium is an essential micronutrient for the proper functioning of animal and human organisms. Thus, fertilization with Se is applied in some countries, as the plants absorbing this element use it as their main dietary source. However, in geographic areas rich in selenium-containing minerals, the excess of Se may be toxic. As a result, the monitoring of water contamination and its accumulation in animal tissues is necessary [23]. The mechanisms of action of Se include the activation of enzymatic and non-enzymatic antioxidants [24], in addition to interference in the transport of micro- and macro-elements taking part in the correct course of metabolic processes [25]. Moreover, the involvement of Se in maintaining the stability of lipid membranes in the presence of hormone substances was indicated by Gzyl-Malcher [26]. As ZEA may be located in membranes [27] due to their hydrophobic–hydrophilic construction and can reveal hormonal functions in plants [28], selenium can also protect membranes against this toxin. On the other hand, brassinosteroids can change the structural properties of membranes. It was shown that participation of steroids in the defense of membranes against the destructive action of amphipathic toxins depended on the localization of polar groups in steroid molecules [29]. As brassinosteroids have a ZEA-like structural relativity, they could eliminate ZEA from the sites where it is usually incorporated into cell membranes.

The aim of the present study was to show whether and to what extent selenium and 24-epibrassinolide (representative of brassinosteroids; EBR) used in studies of the actions of steroids [30] diminish the stressogenic effect of ZEA in wheat seeds. The intensity of stressogenic effects was estimated on the basis of qualitative and quantitative EPR characteristics of changes found for paramagnetic metal species. In the present experiments, the toxic effect of ZEA and the protective role of Se and EBR were examined in grains and germinating seeds of wheat genotypes with various tolerances to oxidative stress. The possibility of the use of EPR in such studies was indicated in our previous experiments [18,31]. The activation of enzymatic antioxidants was determined in order to confirm the ability of investigated genotypes to stimulate protecting mechanisms in the presence of ZEA.

## 2. Results

### 2.1. Zearalenone Content and Antioxidant Activity

Grains of both investigated wheat genotypes contained ZEA in similar and low concentrations, although this was slightly higher in the case of Raweta (Table 1). After a 24-h treatment with the ZEA solution, the accumulation of this toxin increased by about 4.8-fold and 5.0-fold for Parabola and Raweta, respectively. In the mixtures of ZEA + Se and ZEA + EBR, a decrease in ZEA uptake was about 30% and 15% for both genotypes, respectively, when compared to pure ZEA solutions. In the presence of Se and EBR separately, contamination of ZEA occurred at the same level as in the control samples.

In the hypocotyl tissues, ZEA was accumulated in a similar way as in parent grains. Essentially, higher amounts were found in Raweta (about three-fold) compared to Parabola (about two-fold) in comparison to controls. This was diminished by the addition of Se and EBR to the ZEA solution by a smaller percentage in the sensitive genotype (about 20% and about 30% for Raweta and Parabola, respectively) (Table 1).

Analysis of the antioxidative enzymes system indicated that in Parabola hypocotyls, the activities of SOD, POX, and APX were generally higher than in Raweta. This finding was the opposite to CAT, as the activity of this enzyme was lower in the Parabola genotype (Table 2). The presence of ZEA in the solution of germinated grains induced an increase in SOD and CAT activity (especially in tissues of Raweta), and a decrease in activity of POX. In other investigated solutions, activities of these enzymes were close to those of controls. For APX activity, no significant differences between treatments were detected.

### 2.2. Electron Paramagnetic Resonance Spectra

The chemical character of the paramagnetic metal ions and organic radicals present in the control samples of grains and hypocotyls, as well as its changes caused by treatment with ZEA, Se, EBR in addition to mixtures of ZEA + Se and ZEA + EBR was estimated based on the simulation of EPR spectra. This procedure allowed for the determination of quantitative characteristics of these changes, the contribution of individual signals originating from different species to integral spectra intensity, as well as establishing EPR parameters: g-factors and hyperfine splitting values (hfs; A). The g-factor provides information about the electronic structure of the paramagnetic center, because it reflects the influence of local magnetic fields on unpaired electrons by spin–orbit coupling. Therefore, it is possible to determine the nature of the atomic or molecular orbital containing this electron and attribute the EPR signal to the defined paramagnetic individual.

**Figure 1:**
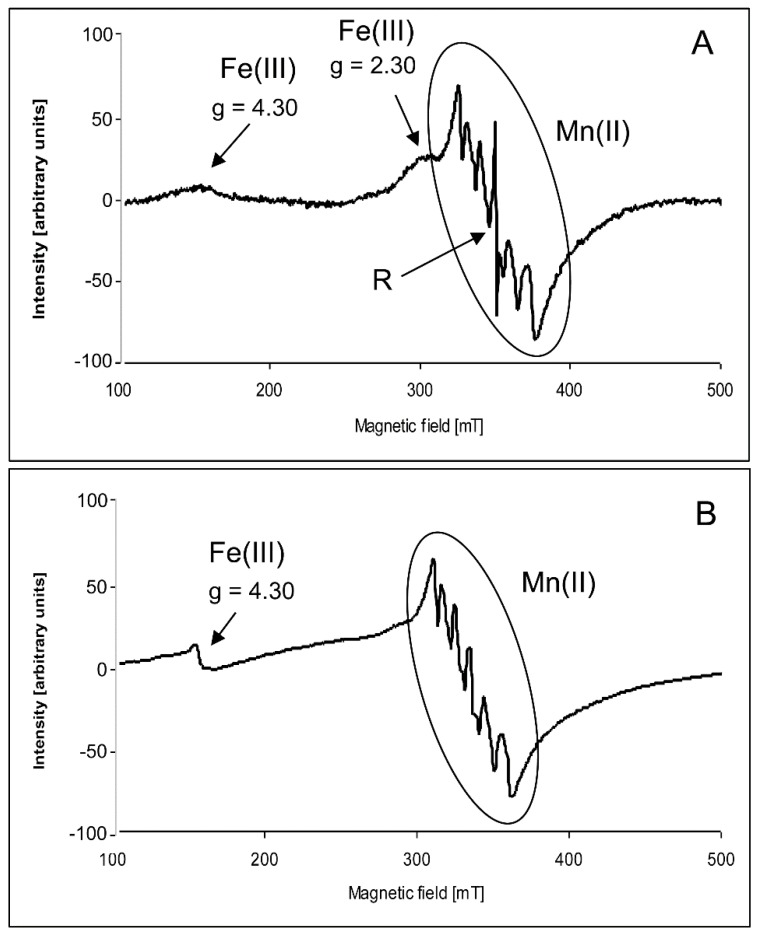
Example of the electron paramagnetic resonance (EPR) spectra of wheat grains (control) registered (**A**) at 293 K and (**B**) at 77 K.

The measurements of grains were performed at 293 K and at 77 K, whereas the spectra of wheat hypocotyls were recorded only at 77 K, because the high water content in this plant material prevented measurements at room temperature. It was found that the control samples of both types of grains exhibited the characteristic signal with six lines of hyperfine structures (hfs) overlapping on a broader line at *g* = 2.00 (attributed to Mn(II)) (Figure 1). Between the third and fourth lines of hyperfine structure, a narrow signal at *g* = 2.00 (organic radicals) was clearly visible. In the spectra, low intensive signals at *g* = 2.30 and *g* = 4.30 were observed. Both signals are attributed to the Fe(III) species [30]. In the spectra registered at 77 K, the signal with the hyperfine structure became more intensive in the spectra of both genotypes. Simultaneously, the narrow signal at *g* = 2.00 broadened and decreased its intensity. The signal at *g* = 2.30 disappeared, whereas the signal at *g* = 4.30 grew (Figure 1B). Under the influence of ZEA, ZEA + EBR, and ZEA + Se, intensities and shapes of the spectra measured at 293 K and 77 K were not substantially altered.

**Figure 2:**
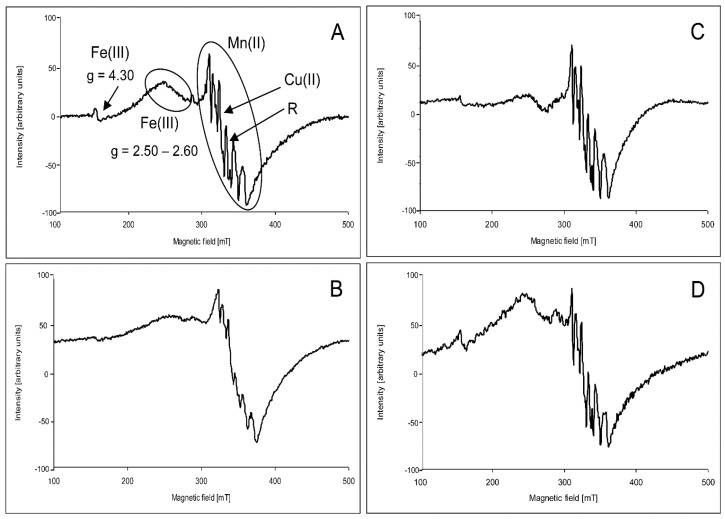
Example of the electron paramagnetic resonance (EPR) spectra of wheat hypocotyl registered at 77 K: (**A**) control; (**B**) after treatment of grains with ZEA; (**C**) after treatment of grains with ZEA + Se; and (**D**) after treatment of grains with ZEA + EBR.

The character of the hypocotyl spectra of both wheat genotypes was very similar to that of the spectra of grains (Figure 2A). The most intensive signal of the spectra was composed of six lines of the hyperfine structure with A = 9.2 mT appearing on a broad line with *g* = 2.00, similar to what was observed in the grain spectra. Furthermore, there was also a narrow signal registered at *g* = 2.00 located between the third and fourth hfs lines. Moreover, the signal at *g* = 2.05 overlapping the third line of the hfs structure could be seen in the spectra. In contrast to the spectra of grains, a broad signal with *g* in the range of 2.50–2.60 appeared in the spectra of hypocotyls. A low intensity signal at *g* = 4.30 was also visible.

**Figure 3:**
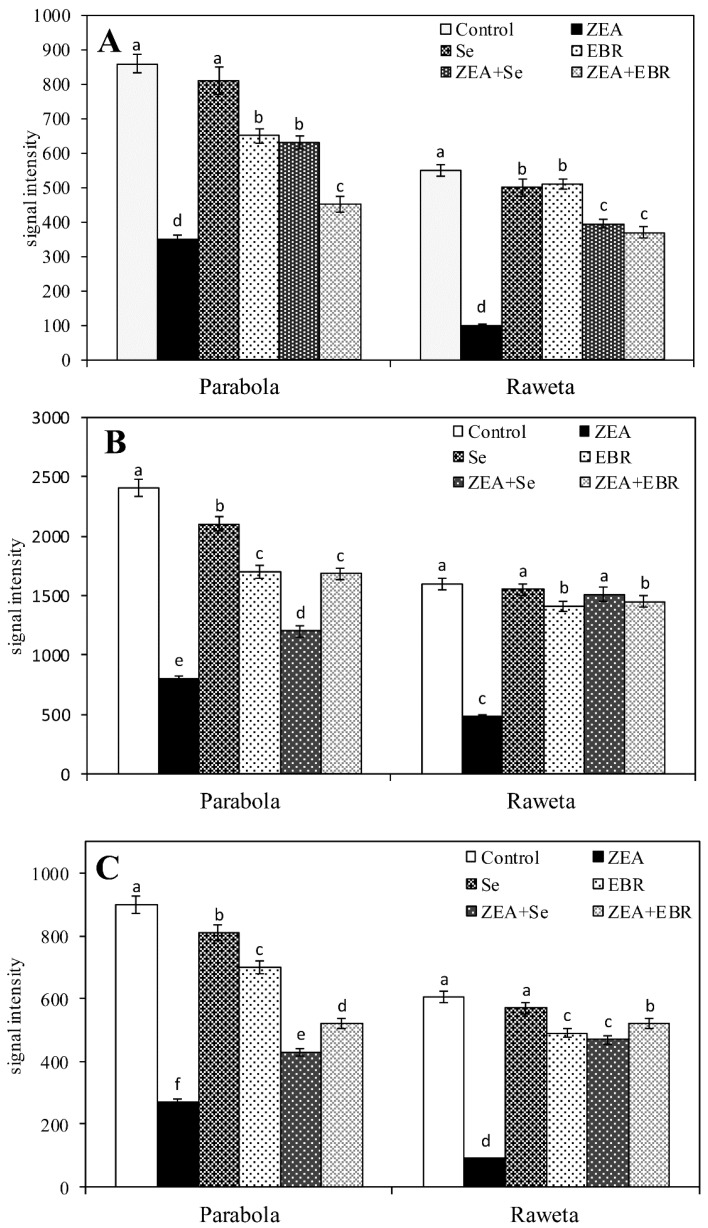
The intensities of EPR signals of transition metal ions in the spectra of Parabola and Raweta hypocotyl controls and treated with various agents: (**A**) signal of Mn(II) ions in aqua complex; (**B**) signal of Mn(II) ions protein complex; and (**C**) signal of Fe(III) ions protein bonded. Data represent the mean from three independent experiments ± standard error (SE). Different letters in the same row indicate significant inter-group differences, *p* ≤ 0.05; the same letters in the same row indicate insignificant inter group differences, *p* ≤ 0.05.

Under the action of ZEA, a broad signal at *g* = 2.50–2.60 strongly decreased, especially in Raweta samples (Figure 2B). In contrast, upon ZEA + Se (Figure 2C) and ZEA + EBR (Figure 2D) treatment, its intensity was slightly lower than that of the control, but higher than that found for a plant treated with ZEA. In Parabola, there was a significantly higher signal intensity in the control spectrum compared to the Raweta one, as ZEA resulted in a smaller decline in the intensity of this signal (Figure 3). The influence of the mixture of ZEA with EBR and ZEA with Se led to a decrease in signal intensities equal to approximately half of the intensity registered for the control samples. Very similar changes were observed for the broad line overlapped by a signal with hyperfine structure. In the Parabola control samples, this signal had an intensity about two times higher than that in the Raweta spectrum. Its changes for both genotypes under the influence of ZEA, ZEA + EBR, and ZEA + Se were similar to those observed for signal at *g* = 2.50–2.60. The intensity of the first line of the hyperfine structure was altered in the same way when the above agents were applied. EBR and Se—acting separately—caused the appearance of signals with intensities similar to those found for control samples in the case of selenium addition and with lower intensities in the case of EBR.

**Figure 4:**
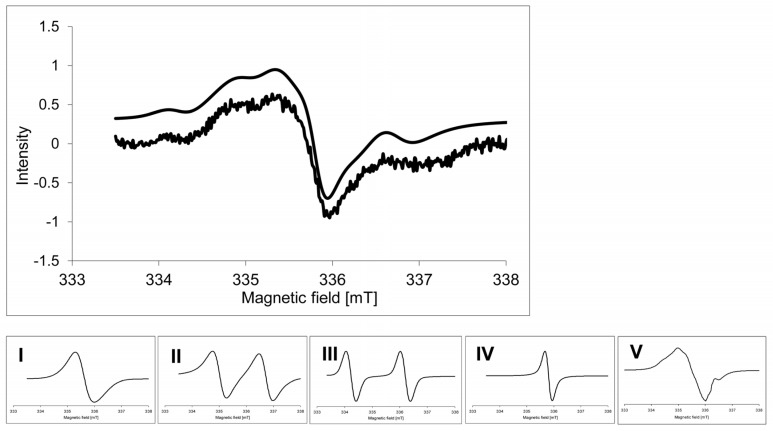
Example of the organic radical signal of wheat hypocotyl (control) registered at 77 K. Signals I–V (presented with normalized intensities) were used for simulation of the spectrum: (**I**) semiquinone radical; (**II**) carbohydrate radical in molecule with low molecular weight; (**III**) carbohydrate radical in molecule with high molecular weight; (**IV**) chlorophyll radical; and (**V**) tyrosyl radical.

In order to record the details of the narrow signal at *g* = 2.00, the EPR spectra of hypocotyls were taken in the range of 5 mT (Figure 4). Simulation of the spectra allowed us to distinguish five signals with the parameters listed in Table 3. It was found that the radical signal intensity slightly increased upon ZEA treatment, and was found to be stronger in Raweta. The addition of EBR and Se to the ZEA solution led to a decrease and broadening of the signal, which had an intensity that was difficult to estimate. The action of the ZEA + EBR mixture increased the intensity of the spectra, reaching a level of control in the Parabola genotype, while treatment with ZEA + Se led to spectra with intensities similar to those recorded for samples with only ZEA added. The addition of EBR caused the appearance of a signal with an intensity comparable to that observed for control, whereas the signal intensity was lower with the addition of Se.

## 3. Discussion

Despite the lack of visually observed changes on the surface of the seeds, UHPLC showed that grains of the investigated genotypes contained ZEA, although this was found in rather low levels. As the grains originated from plants cultivated in the same year and in the same region, the ZEA concentration was similar in both tolerant (Parabola) and sensitive (Raweta) control wheat samples. Exogenous application of the ZEA solution caused an increase in its accumulation in grains and the possibility of infecting the germinated plants with this toxin. A delay in the germination of grains was the physiological consequence of ZEA treatment (from 48 h for controls to 58 h for ZEA-treated grains). The stronger effect observed for the sensitive Raweta genotype was most likely connected with the higher accumulation of ZEA in these plants.

These are the first results describing the relationship between the amount of zearalenone absorbed by grains and the amount of this toxin accumulating in the developing plants of the stress-tolerant and stress-sensitive genotypes.

In comparison with Parabola, the possibility of a stronger stimulation of oxidative stress via ZEA treatment was confirmed in the sensitive genotype by the observed activation of antioxidative enzymes—especially SOD (responsible for removing of superoxide radical) and CAT (involved in the decomposition of H_2_O_2_) [15]. POX and APX appeared to be less involved in the deactivation of ROS generated during the stress actions of ZEA. The protective substances used in this study (namely Se ions and EBR) were found to decrease ZEA accumulation in grains and hypocotyls at a higher degree in the stress-tolerant wheat, which was also observed to have smaller effects of ZEA-stimulation of the activity of antioxidative enzymes.

EPR studies have shown: (i) that in the mechanism of antioxidants stimulated in the presence of zearalenone, the paramagnetic metal ions and organic radicals bound into biomolecules are also involved, in addition to the antioxidant enzyme systems and (ii) to what extent the presence of EBR and Se modifies this mechanism. Based on the conducted analyses, it was found that the paramagnetic metal ions are more engaged in the process of deactivating the excess electrons generated under conditions of the zearalenone oxidative stress than organic radicals.

The EPR spectroscopy provided information on the changes in the amount and character of Mn(II) and Fe(III) species occurring as a result of ZEA treatment. The main signal of all spectra was composed of a broad line with overlapping well-resolved hyperfine structure, which was attributed to the manganese species. The hyperfine structure originated from the freely rotating aqua-complex of Mn(II), and was often detected in the EPR spectra of plant materials [19,31,32]. The broad line was attributed to dipole–dipole interacting manganese ions situated in the protein matrix. A thorough discussion of Mn(II) signals and their attribution to particular Mn species was made in our previous paper [31].

In hypocotyl samples investigated in this work, the signal of the Mn(II) aqua complex exhibited higher intensity in the spectra of tolerant genotype, and was found to be decreased after ZEA (Figure 3A). This might be caused by disintegration of the structure of the [Mn(H_2_O)_6_]^2+^ complex, which was found to be more advanced in the sensitive genotype than in the tolerant one. Subsequently, this points to a higher stability of the manganese aqua complex in the latter. The signal of Mn–protein complexes also exhibited a higher intensity in the spectra of the tolerant genotype, which was decreased after ZEA treatment and was stronger in the case of the sensitive one. Thus, this indicates greater damage of protein structures in the latter (Figure 3B). The broad line at *g* = 2.50–2.60 could be ascribed to Fe(III) ions bonded to the protein in the ferritin shell [33,34,35]. Similar to the signal of the Mn(II)–protein complex, this line was also more intensive in the tolerant genotype and decreased after ZEA treatment. As this effect was more noticeable in the sensitive genotype, this pointed to a stronger susceptibility of the latter genotype to damage of the protein-complex structure after ZEA treatment (Figure 3C). Another signal of an iron species was observed in all spectra at 77 K with *g* = 4.30, and was attributed to the non-haem, high spin Fe(III) with rhombic symmetry [20,32]. In some spectra, a line at *g* = 2.05 could also be seen. Tentatively, this signal might be described as a perpendicular *g* component of the signal of copper ions in the square planar complexes of proteins [32,36].

The changes observed during our experiment could suggest a strong negative influence of ZEA on the stability of the Fe–protein and Mn–protein complexes, which are important in vital processes. More intensive changes in the Fe(III) and Mn(II) signals after ZEA treatment were observed in the spectra of the sensitive genotype. The intensity of manganese signal with hfs in the Parabola control and the change after the addition of particular agents indicated that the higher concentration of manganese aqua-complexes as well as their smaller reduction after ZEA in comparison to Raweta was evidence of better water accumulation in tissues of the tolerant genotype.

The observed influence of ZEA + EBR treatment on plants suggested an inhibition of ZEA action by EBR. A similar effect was caused by the addition of Se, indicating that both selenium and EBR were the powerful agents to defend plants against the influence of ZEA.

The narrow line at *g* = 2.00—often found in spectra of different plant materials—was assigned to organic radical species [18,19,37]. The simulation procedure allowed us to distinguish the five signals building this line (Figure 4, Table 3). Signals I and V were ascribed to the phenoxyl species. Signal I exhibited parameters typical for semiquinone radicals [38,39,40]. Signal V with the highest contribution to the spectrum could be attributed to the tyrosyl radical, as it presented a slightly higher anisotropy than signal I and characteristic hyperfine structure [12,31]. These parameters suggest that it was a radical formed naturally during life processes involving redox reactions [37,41,42,43] and was detected in the PSII photosynthetic system [44]. Signals II and III originated from carbon-centered radicals: signal II with a lower *g* value was attributed to carbohydrate radicals formed in molecules with a low molecular weight [45,46], whereas signal III with a higher *g* value was assigned to radical species located in carbohydrate molecules with a high molecular weight (polysaccharides) [19,47]. The parameters of the narrow signal IV pointed to its origin from the chlorophyll radical cation [32]. The increasing intensity of spectra of radicals observed after ZEA treatment confirmed that there was destruction of organic matrices stabilizing these radical species, which is in agreement with changes in the biochemical surroundings of transition metal ions. The disturbance of organic material in both cases was more noticeable in the sensitive genotype.

## 4. Conclusions

These studies showed that ZEA introduced to seeds appeared in large quantities in germinating seedlings (hypocotyls) of wheat. Selenium ions and 24-epibrasinolide reduced the intake of ZEA in the grains, and consequently decreased the amount of this toxin in the seedlings. Oxidative stress induced under ZEA treatment influenced the activation of the antioxidative enzymes, which was found mainly in the sensitive genotype. EPR measurements allowed us to characterize the effects of ZEA action on the stability of the organic matrix. In the tolerant genotype, there was a lower degree of changes related to injuries mainly of protein structures compared with the sensitive one. The use of protective substances introduced together with ZEA into grains inhibited the process of the destruction of biochemical structures. However, individually applied protective substances did not influence or decrease the intensity of signals in comparison to controls.

## 5. Material and Methods

### 5.1. Plant Material

Grains of spring wheat (*Triticum aestivum*)—namely Parabola (tolerant) and Raweta (sensitive)—were obtained from the Polish Wheat Breeding Institute in Kobierzyce (Poland) from the crops cultured in 2015. The tolerance of both genotypes to oxidative stress was confirmed in earlier experiments [48]. No sprouting injury or browning was observed in any of the samples studied. Before analysis, the surface of grains was sterilized with 90% ethanol (1 min) and then rinsed five times with distilled water. Samples of 100 grains in three replicates were collected in petri dishes for each treatment: control (distilled water), ZEA (30 µM), Se (5 µM Na_2_SeO_4_), EBR (0.1 µM), as well as ZEA + Se and ZEA + EBR. Measurements of the zearalenone accumulation and EPR analyses were performed for swollen grains after 24 h of treatment in used solutions. This was repeated for hypocotyl fragments of 1 cm length, which were cut off from germinated seeds (48–58 h of treatment in used solutions). Time differences were caused by various speeds of seed germination in ZEA solutions to obtain hypocotyls of 1 cm long. The activities of the antioxidative enzymes were determined in the samples of 1 cm fragments of hypocotyl, which were frozen in liquid N_2_ and maintained at a temperature of −80 °C.

### 5.2. Chemicals

ZEA was purchased from Fermentek (Jerusalem, Israel). Other chemicals used in this study were purchased from Merck (Darmstadt, Germany).

### 5.3. Determination of Zearalenone Concentration

ZEA content was determined by the UHPLC (Infinity 1260, Agilent Technologies, Santa Clara, California, USA) technique with a tandem quadruple mass spectrometry detector (QQQ 6410). Before analysis, samples of grains (2.5 g) and hypocotyls (2 g) were ground into powder and extracted with acetonitrile/water (90:10, *v/v*) according to Gromadzka et al. [49], with modifications per the findings of Luo et al. [50]. The extract was shaken for 1 h on a rotary shaker (RL-2020, JW_Electronic, Warsaw, Poland), and after filtration it was purified on a Bond Elut Mycotoxin column (45 mm/1000 mg, part No. 12165001B, Agilent Technologies, Santa Clara,, California, USA). A total of 10 μL of extracts was injected to the analytical column (3.0 × 100 mm, 2.7 µm, LN B10006, Poroshell 120 Phenyl-Hexyl). As a mobile phase, a mixture of acetonitrile/water/methanol (46:46:8, *v/v/v*) at a flow rate of 0.3 mL/min and a temperature of 30 °C was used. The MS source parameters were a gas temperature of 350 °C and a capillary voltage of 4000 V. A quadruple mass analyzer in the mode of monitoring ions with a positive ratio of atomic mass to charge (*m*/*z* = 317.2) was used.

### 5.4. Analysis of Antioxidative Enzymes

Frozen hypocotyl tissues (about 200 mg) were homogenized in 100 mm of potassium phosphate buffer (containing 2 mm of a-dithiothreitol, 0.1 mm of EDTA, and 1.25 mm of polyethylene glycol at a pH of 7.8). After filtration and centrifugation (14,000 *g* for 30 min at 4 °C), the supernatant was collected for analysis of antioxidant enzymes as described in a previous study [38]. The activity of superoxide dismutase (SOD; EC 1.15.1.1) was determined by measuring cytochrome reduction assay at 550 nm. One unit of SOD was defined as the amount of enzyme that inhibited the rate of cytochrome reduction by 50% [51]. Catalase (CAT; EC 1.11.1.6) activity was measured by a decrease in H_2_O_2_ content at 240 nm [52]. Peroxidases (POX; EC 1.11.1.7) were determined as the amount of products from a 1% p-phenylenediamine reaction with 0.03 mm H_2_O_2_ at 485 nm [53]. The activity of ascorbic peroxidase (APX; EC 1.11.1.11) was assigned as the decrease in absorbance at 290 nm of an assay mixture containing 0.5 mm of ascorbate [54]. The reaction progress of all enzymes was examined 2 min after the initiation of the reaction and registered using KINLAB software. Enzyme activities were expressed relative to protein content in the supernatant.

### 5.5. Electron Paramagnetic Resonance Spectroscopy

The Bruker ELEXSYS 500 spectrometer (Karlsruhe, Germany) with a 100 kHz field modulation was used to register the EPR spectra of plant material. The spectra were recorded in the X-band (~9 GHz) at 293 K and at 77 K in the range of 5 mT and 500 mT with a microwave power of 3 mW and 10 mW, respectively. The spectra of three samples of each plant specimen were registered in duplicate. 1,1-Diphenyl-2-picrylhydrazyl (DPPH) was used for the g-factor standard. The intensity of the particular signal or the integral intensity of EPR spectrum, related to 1 g of the sample, was used as a measure of the number of paramagnetic centers in the studied material.

Simulation of radical spectra was performed using the modified program SIM 32 [55]. In this procedure, the theoretical spectrum was the sum of signals assigned to particular paramagnetic centers in the assumed model. The method allowed us to acquire contributions and EPR parameters such as *g* factors and A splitting constants for all the signal components of the spectrum.

### 5.6. Statistical Analysis

The biological data were reported as means of triplicate analyses (± SE). Differences were analyzed using one-way ANOVA, followed by post-hoc comparisons using Statistica Software version 9.1 (SAS Institute Inc., Cary, NC, USA). Variations between samples versus controls were considered significant for *p* < 0.05.

The EPR parameters were calculated with the accuracy of ±0.0002 for *g* values and ±0.05 mT for hyperfine splitting constant A of radicals. The contribution of particular signals was the average of three simulations of spectra for each sample.

## Figures and Tables

**Table 1:** Content of zearalenone (ZEA) in grains analyzed after 24 h treatment (grains) and after 48–58 h treatments (hypocotyls cut off from germinated grains) with 30 µM ZEA, 10 µM Na_2_SeO_4_ (Se), 0.1 µM 24-epibrassinolide (EBR), and of mixtures of ZEA + Se and ZEA + EBR.

Genotype	ZEA (ng/g)		Genotype	ZEA (ng/g)	
Parabola	grains	hypocotyl	Raweta	grains	hypocotyl
control	60.8 ± 0.9 ^c^	45.2 ± 1.0 ^c^	control	70.4 ± 1.4 ^c^	52.7 ± 1.2 ^c^
ZEA (30 µM)	294.7 ± 2.3 ^a^	101.3 ± 2.6 ^a^	ZEA (30 µM)	350.0 ± 3.9 ^a^	155.4 ± 2.0 ^a^
Se (10 µM)	59.9 ± 1.3 ^c^	44.1 ± 1.1 ^c^	Se (10 µM)	70.3 ± 1.6 ^c^	53.6 ± 1.5 ^c^
EBR (0.1 µM)	59.5 ± 1.1 ^c^	45.0 ± 1.7 ^c^	EBR (0.1 µM)	70.8 ± 1.7 ^c^	54.0 ± 1.4 ^c^
ZEA + Se	205.6 ± 2.6 ^b^	81.5 ± 2.0 ^b^	ZEA+Se	302.1 ± 2.7 ^b^	128.3 ± 3.9 ^b^
ZEA + EBR	199.1 ± 3.8 ^b^	76.6 ± 2.2 ^b^	ZEA + EBR	297.0 ± 3.0 ^b^	126.5± 2.6 ^b^

Data represent the mean from three independent experiments ± standard error (SE). Different letters in the same row indicate significant inter-group differences, *p* ≤ 0.05; the same letters in the same row indicate insignificant inter group differences, *p* ≤ 0.05.

**Table 2:** Antioxidative enzymes (SOD—superoxide dismutase, CAT—catalase, POX—peroxidases and APX—ascorbic peroxidase) in hypocotyls germinating on the Parabola and Raweta wheat grains in solutions of 30 µM of ZEA, 10 µM Na_2_SeO_4_ (Se), 0.1 µM 24-epibrassinolide (EBR), and of mixtures of ZEA + Se and ZEA + EBR.

Treatment	Antioxidative Enzymes (U/mg Proteins)			
	SOD	CAT	POX	APX
*Parabola*				
control	0.29 ± 0.01 ^a^	0.039 ± 0.002 ^a^	0.37 ± 0.02 ^a^	0.019±0.004 ^a^
ZEA (30 µM)	0.31 ± 0.02 ^a^	0.042 ± 0.002 ^a^	0.34 ± 0.03 ^a^	0.018±0.002 ^a^
Se (10 µM)	0.25 ± 0.02 ^ab^	0.035 ± 0.002 ^ab^	0.36 ± 0.01 ^a^	0.016 ± 0.003 ^a^
EBR (0.1 µM)	0.26 ± 0.02 ^ab^	0.039 ± 0.001 ^ab^	0.39 ± 0.02 ^a^	0.017 ± 0.003 ^a^
ZEA + Se	0.28 ± 0.02 ^a^	0.040 ± 0.002 ^a^	0.36 ± 0.03 ^a^	0.016 ± 0.004 ^a^
ZEA + EBR	0.29 ± 0.01 ^ab^	0.037 ± 0.003 ^a^	0.36 ± 0.01 ^ab^	0.017 ± 0.003 ^a^
*Raweta*				
control	0.12 ± 0.02 ^b^	0.047 ± 0.001 ^b^	0.30 ± 0.01 ^a^	0.007 ± 0.003 ^a^
ZEA (30 µM)	0.25 ± 0.03 ^a^	0.053 ± 0.001 ^a^	0.25 ± 0.01 ^b^	0.010 ± 0.003 ^a^
Se (10 µM)	0.14 ± 0.01 ^b^	0.048 ± 0.003 ^b^	0.29 ± 0.02 ^a^	0.008 ± 0.004 ^a^
EBR (0.1 µM)	0.14 ± 0.02 ^b^	0.043 ± 0.004 ^b^	0.26 ± 0.02 ^ab^	0.006 ± 0.002 ^a^
ZEA + Se	0.16 ± 0.02 ^b^	0.045 ± 0.003 ^b^	0.26 ± 0.02 ^ab^	0.011 ± 0.004 ^a^
ZEA + EBR	0.15 ± 0.03 ^b^	0.047 ± 0.002 ^ab^	0.27 ± 0.03 ^ab^	0.011 ± 0.004 ^a^

Data represent the mean from three independent experiments ± standard error (SE). Different letters in the same row indicate significant inter-group differences, *p* ≤ 0.05; the same letters in the same row indicate insignificant inter-group differences, *p* ≤ 0.05.

**Table 3:** The parameters of electron paramagnetic resonance (EPR) signals of radicals and their contributions to the spectra (mean values).

Signal	I	II	III	IV	V
*g*	*g*_1_ = 2.0058 *g*_2_ = 2.0041 *g*_3_ = 2.0013 *g*_av_ = 2.0037	2.0023	2.0062	2.0027	*g*_1_ = 2.0078 *g*_2_ = 2.0045 *g*_3_ = 2.0023 *g*_av_ = 2.0049
A (mT)	-	1.7	2.0	-	A_2_^β^ = 1.7
Contribution (%)	20	14	4	28	34
